# Veterinary Register

**Published:** 1901-11

**Authors:** 


### VETERINARY REGISTER.

[See Editorial, Journal of Comparative Medicine and Veterinary Archives, July, 1901, page 438.]

[In the following list the names and data printed without comment are those furnished to us by postal card with the signature of the individual.

Those with an asterisk [*] are those to whom we have sent, by United States mail, an envelope with prepaid postage and a request for return if not delivered in ten days; as the envelopes have not been returned, we presume that the letter has been delivered, that the individual in question exists, but that he has neglected to return the enclosed postal card with the information requested.—Ed. Journal.]

### PENNSYLVANIA.

(Continued from page 682.)

| *Name.* | *Street.* | *Town.* | *College, Graduated.* | *County, Registered.* |
|---|---|---|---|---|
| Ickes, Geo. H. | ...... | King | * | ...... |
| Irons, J. B. | 1230 Peach st | Erie | Ontario Vet., 1884 | Crawford, 1889 |
| Jackson, A. W. | ...... | Mercer | * | ..... |
| Jackson, H. B. | ...... | Sewickley | * | ...... |
| Jackson, L. E. | ...... | Hadley | * | ...... |
| Jacob, Casper | ...... | Bakersville | * | ...... |
| Jacoby, F. H. | ...... | Litzenberg | * | ...... |
| Jacoby, Wm. | ...... | Dreibelbis | * | ...... |
| Jacquett, Samuel P. | ...... | Radnor Twp. | * | ...... |
| James, D. J. | 1306 Race st | Philadelphia | * | ...... |
| Jarvin, P. | ...... | Kane | * | ...... |
| Jeffries, Jos. R. | 1725 Arch st | Philadelphia | * | ...... |
| Jennings, Robert, Jr. | ...... | Pittsburg | * | ...... |
| Jervis, F. C. | ...... | Edinboro | Ontario Vet., 1892 | Crawford, 1891, Mercer, 1893 |
| Jobson, George | ...... | Oil City | * | ...... |
| Jobson, George B. | 1122 Liberty st | Franklin | * | ...... |
| Jobson, John A. | ...... | Franklin | * | ...... |
| Jobson, W. R. | Elk st | Franklin | Nat. Vet. Surg., 1896 | Venango, 1896 |
| Joerg, E. F. C. | ...... | Coudersport | * | ...... |
| Johns, Edwin M. | ...... | Morristown | * | ...... |
| Johnson, Charles | ...... | Lansdale | * | ...... |
| Johnson, Joseph | ...... | Kirkwood | V. D. U. Pa., 1898 | Lancaster |
| Johnson, Samuel H. | 1834 N. 11th st | Philadelphia | * | ...... |
| Johnson, Walter S. | ... .. | Moores | * | ...... |
| Johnston, H. J. | ...... | Canton | * | ...... |
| Jones, Lindsey | ...... | Simpson's Store | * | ...... |
| Jones, John | ...... | Tremont | * | ...... |
| Jones, Robert | ...... | Apollo | * | ...... |
| Jones, W. A. | ...... | Millerstown | * | ...... |
| Jones, W. H. | ...... | Millerstown | * | ...... |
| Kaffroath, Geo. B. | ...... | Ephrata | * | ...... |
| Kahle, F. M. | ...... | Shippensville | * | ...... |
| Kahle, F. P. | ...... | Franklin | * | ...... |
| Kahle, Thos. M. | ...... | Shippensville | * | Clarion, Venango, 1889; Forest, 1898 |
| Kain, F. M. | 118 N. George | York | American Vet., 1883 | York |
| Kame, W. J. | Webster av | Pittsburg | * | ...... |
| Kann, R. L. | ...... | Lisburn | * | ...... |
| Kapp, Geo. F. | ...... | Fryburg | * | ...... |
| Kauffman, H. F. | 1737 Center st | Ashland | Ontario Vet., 1889 U.S.V.S., 1900 | Schuylkill, 1900 |
| Kauffman, Sol. W. | ...... | Vanwert | * | ...... |
| Kauffman, Wm. | ...... | Zelienople | * | ...... |
| Kaul, Wm. | ...... | St. Mary's | * | ...... |
| Kean, T. J. | 1534 N. Carlisle | Philadelphia | V.D. U. Pa., 1890 | Clinton, 1890 |

| *Name.* | *Street.* | *Town.* | *College, Graduated.* | *County, Registered.* |
|---|---|---|---|---|
| Keck, Morris W. | 520 Main st | Slatington | Ontario Vet., 1888 | Lehigh, 1889 |
| Keech, James | ...... | Embreeville | * | ...... |
| Keefer, Jeremiah | ...... | E. Hanover Twp. | * | ...... |
| Keeler, H. B. | ...... | Kulpsville | * | ...... |
| Keeler, Jesse H. | ...... | Kulpsville | * | ...... |
| Keelor, Allen Z. | ...... | Harleysville | * | ...... |
| Keelor, J. Rein | ...... | Harleysville | Ontario Vet., 1883 | Montgomery |
| Keely, Horace P. | ...... | Schwenkville | V.D. U. Pa., 1892 | Montgomery, 1892 |
| Keener, J. B. | ...... | Slate Lick | * | ...... |
| Keeney, Fred E. | ...... | W. Hanover | * | ...... |
| Keil, Z. S. | ...... | Perkasie | * | ...... |
| Keim, S. C. | 17 N. Linden | Bethlehem | * | ...... |
| Keister, Henry | ...... | Somerset | * | ...... |
| Keiter, C. F. | ...... | Williamstown | * | ...... |
| Keith, Thos. | ...... | Martinsburg | * | ...... |
| Keller, David M. | 514 Market st | Williamsport | Ontario Vet., 1886 | Lycoming |
| Kellner, Edward K. | 1621 Brown st | Philadelphia | * | ...... |
| Kelly, A. F. | ...... | Gazzam | * | ...... |
| Kelly, Frank | ...... | Fairfield Twp. | * | ...... |
| Kelso, John | ...... | Lurgan | * | ...... |
| Kendig, Alfred | ...... | Conestoga | * | ...... |
| Kendig, M. H. | ...... | Conestoga | * | ...... |
| Kennedy, John | 4324 West'nster | Philadelphia | * | ...... |
| Kennedy, M. M. | 1532 N. 13th st | Philadelphia | * | ...... |
| Kennedy, Wilbur | ...... | Pleasant Mt. | * | ...... |
| Kent, Z B. | 214 Strawb'y | Harrisburg | * | ...... |
| Kern, Josiah | ...... | Orefield | * | ...... |
| Keyser, D. P. | 1838 Arch st | Philadelphia | * | ...... |
| Kiethline, J. T. | Courtland st | E. Stroudsb'g | Ontario Vet., 1895 | Wyoming, 1895 |
| Kime, John T. | ...... | Grant | * | ...... |
| Kimmel, David E. | ...... | Brownsville | * | ...... |
| King, Green B. | ...... | Scullton | * | ...... |
| King, Ross R. | ...... | New Lex'gton | * | ...... |
| Kingan, J. C. | 4062 Liberty av | Pittsburg | Ontario Vet., 1896 | Allegheny, 1897 |
| Kingsland, John C. | Second st | Canton | N. Y. C. V. S., 1895 | Bradford, Pa., & Chemung, N. Y., 1895 |
| Kinter, Jos. B. | ...... | Marion Centre | * | ...... |
| Kipe, Joseph | ...... | Williamsburg | * | ...... |
| Kirk, Theo. T. B. | ...... | Rushland | * | ...... |
| Kirk, Wm. J. | ...... | Sharon | * | ...... |
| Kistler, A. H. | ...... | Jefferson Twp. | * | ...... |
| Kistler, A. H. | 248-50 W. Jeff'n | Butler | Ontario Vet., 1894 | Butler |
| Kline, Harry E. | ...... | York | * | ...... |
| Kline, J. J. | 17 E. Market st | Danville | Ontario Vet., 1887 | Montour |
| Knapp, Ira | ...... | Austinville | * | ...... |
| Knight, L. J. | ...... | Harrison Val. | * | ...... |
| Knight, W. H. | Broad st | Kennett Sq. | Philadelphia, 1866 | Chester, 1889 |
| Knoll, L. P. | ...... | Montrose | * | ...... |
| Knoll, L. P., Jr. | ...... | Montrose | * | ...... |
| Knoll, W. L. P. | ...... | Montrose | * | ...... |
| Koehler, Edward F. | 6th & Vine sts | Easton | * | ...... |
| Koehler, Richard A. | ...... | Bethlehem | * | ...... |
| Koehler, Theo. J. | ...... | Fullerton | * | ...... |
| Koehler, Theo. J. | ...... | Easton | * | ...... |
| Koenig, A. O. | 261 S. 15th st | Philadelphia | * | ...... |
| Kohler, Daniel R. | Reading av. | Boyertown | Ontario Vet., 1893 | Berks, 1894 |
| Kolb, Joseph | ...... | Belfast | * | ...... |
| Koller, O. L. | Railroad st | Myerstown | ...... | Lebanon, 1888 |
| Kooker, W. S. | 1021 Buttonw'd | Philadelphia | ...... | Philadelphia |
| Korb, A. F. | ...... | Venus | * | ...... |
| Krater, Adam O. | ...... | Allen Twp. | * | ...... |
| Kreibel, E. G. | ...... | Worcester | * | ...... |
| Kuder, E. E. | 8 E. Pine st | Mahanoy City | ...... | Schuylkill, 1892 |
| Kuhn, J. H. | ...... | N. Mahoning Twp. | * | ...... |
| Kuhn, John M. | ...... | Mercersburg | * | ...... |
| Kuhn, Z. M. | ...... | Mercersburg | * | ...... |

| *Name.* | *Street.* | *Town.* | *College, Graduated.* | *County, Registered.* |
|---|---|---|---|---|
| Kuhns, Wm. H. | ...... | Quakertown | * | ...... |
| Laberge, C. Z. | ...... | Beaver Falls | * | ...... |
| Lacey, J. E. | 16 N. Franklin | Titusville | * | ...... |
| Lacock, J. Stewart | 20 N. Diam'd | Allegheny | V. D. U. Pa., 1895 | Allegheny, 1895 |
| Land, David Cassel | ...... | Gwynedd | ...... | Montgomery, 1889 |
| Landis, H. W. | ...... | Allen | * | ...... |
| Landis, Isaac R. | ...... | Manheim | * | ...... |
| Landis, Tobias E. | ...... | New Berlinville | * | ...... |
| Lantz, M. B. | ...... | Sabula | * | ...... |
| Larrabee, W. H. | ...... | Jackson | * | ...... |
| Larrabee, W. W. | ...... | Susquehanna | * | ...... |
| Larselere, S. D. | ...... | Jenkintown | * | ...... |
| Latshaw, Jonathan | ...... | Schoeneck | * | ...... |
| Layton, Thomas | ...... | Layton | * | ...... |
| Lazarus, Newton C. | ...... | Nazareth | * | ...... |
| Leamer, J. C. | ...... | Canoe Creek | * | ...... |
| Leber, J. G. | 259 E. Main st | Ephrata | * | ...... |
| Leber, Jacob G. | ...... | Mountville | * | ...... |
| Leberman, H. L. | ...... | Meadville | * | ...... |
| Lee, James A. | 708 Cumberl'd | Philadelphia | * | ...... |
| Leeser, Philip | ...... | Sell's Station | * | ...... |
| Lehman, Amos B. | ...... | Fayetteville | * | ...... |
| Lehman, John W. | ...... | Loganville | * | ...... |
| Lehman, Joseph B. | ...... | Hamilton Twp. | * | ...... |
| Lehman, P. A. | 534 W. Phila. st | York | V. D. U. Pa., 1900 | York, 1890 |
| Leinhardt, R. P. | ...... | Wayne | * | ...... |
| Leister, Geo. W. | ...... | Cocolames | * | ...... |
| Lenhart, M. P. | ...... | Circleville | * | ...... |
| Lenker, Simon | ...... | Freeburg | * | ...... |
| Leonard, Chas. | ...... | Dover | * | ...... |
| Lerew, Adam | ...... | Latimore | * | ...... |
| Lesher, Abram | ...... | Chambersburg | * | ...... |
| Lesher, John L. | ...... | Chambersburg | * | ...... |
| Leutholt, Henry | Main st | Taylor | Chicago Vet., 1895 | Lackawanna, 1895 |
| Levi, Moses | ...... | Bellefonte | * | ...... |
| Light, Daniel K. | ...... | Palmyra | * | ...... |
| Lilley Peter | ...... | Schuyler | * | ...... |
| Lintz, Chas, | 1103 Madison | Chester | * | ... .. |
| Lirchenwald, Jas. | ...... | Seipstown | * | ...... |
| Little, Amos | ...... | Laporte Twp. | * | ...... |
| Loftus, James P. | ...... | Carbondale | * | ...... |
| Loller, Jesse G. | Route 3 | Kennett Sq. | * | Chester, 1889 |
| Long, Emanuel W. | ...... | Sporting Hill | * | ...... |
| Longacre, Edwin D. | Main & Coal sts | Shenandoah | Ontario Vet., 1893 | Schuylkill, 1893 |
| Longacre, W. S. | ...... | Mantz | * | ...... |
| Longenecker, Wm. | 112 Main st | Lititz | Ontario Vet., 1893 | Lancaster, 1894 |
| Loy, Jacob E. | ...... | Hampden Twp. | * | ...... |
| Lupher, A. J. | ...... | Townville | * | ...... |
| Lupher, Wm. A. | ...... | Randolph Twp. | * | ...... |
| Lutz, Nathaniel P. | ...... | Tulpehocken | * | ...... |
| Lydick, Thos. B. | ...... | Utah | * | ...... |
| Lydie, B. F. | ...... | Plumville | * | ...... |
| Lyster, E. W. | ..... | Berrysburg | * | ...... |

(*To be continued.*)

